# 4,5-Dibromo-1,2-dimethyl-1*H*-imidazol-3-ium bromide

**DOI:** 10.1107/S1600536812015310

**Published:** 2012-04-13

**Authors:** Mebarek Bahnous, Abdelmalek Bouraiou, Sofiane Bouacida, Thierry Roisnel, Ali Belfaitah

**Affiliations:** a Unité de Recherche de Chimie de l’Environnement et Moléculaire Structurale, CHEMS, Université Mentouri-Constantine, 25000 Algeria; b Laboratoire des Produits Naturels d’Origine Végétale et de Synthèse Organique, PHYSYNOR, Université Mentouri-Constantine, 25000 Constantine, Algeria; c Centre de Difractométrie X, UMR 6226 CNRS Unité Sciences Chimiques de Rennes, Université de Rennes I, 263 Avenue du Général Leclerc, 35042 Rennes, France

## Abstract

In the title salt, C_5_H_7_Br_2_N_2_^+^·Br^−^, the cation and anion are connected by an N—H⋯Br hydrogen bond. In the crystal, there are inter­calated layers parallel to (10-2) in which bromide ions are located between the cations. Weak inter­molecular C—H⋯Br hydrogen bonds are also observed.

## Related literature

For the preparation of the title compound using the Ortoleva–King reaction, see: King (1944 ▶). For applications of *C*,*N*-substituted haloimidazole derivatives, see: Reepmeyer *et al.* (1975 ▶); Zamora *et al.* (2003 ▶); Schmidt & Schieffer (2003 ▶); Mashkovskii (2005 ▶); Amini *et al.* (2007 ▶).
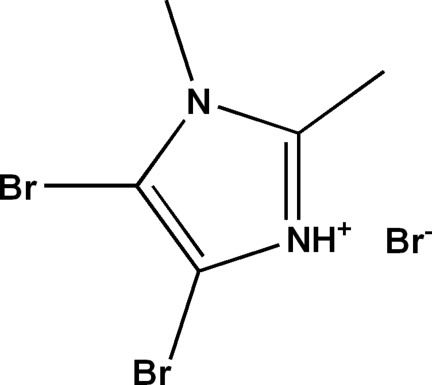


## Experimental

### 

#### Crystal data

C_5_H_7_Br_2_N_2_^+^·Br^−^
*M* *_r_* = 334.86
Monoclinic, 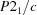

*a* = 5.5938 (3) Å
*b* = 11.2522 (6) Å
*c* = 14.4864 (9) Å
β = 104.571 (3)°
*V* = 882.48 (9) Å^3^
*Z* = 4
Mo *K*α radiation
μ = 13.64 mm^−1^
*T* = 150 K
0.31 × 0.22 × 0.17 mm

#### Data collection

Bruker APEXII diffractometer
Absorption correction: multi-scan (*SADABS*; Bruker, 2002 ▶) *T*_min_ = 0.058, *T*_max_ = 0.098
7565 measured reflections
2032 independent reflections
1747 reflections with *I* > 2σ(*I*)
*R*_int_ = 0.050

#### Refinement

*R*[*F* ^2^ > 2σ(*F* ^2^)] = 0.027
*wR*(*F* ^2^) = 0.060
*S* = 1.03
2032 reflections
94 parameters
H-atom parameters constrained
Δρ_max_ = 0.63 e Å^−3^
Δρ_min_ = −0.86 e Å^−3^

### 

Data collection: *APEX2* (Bruker, 2004 ▶); cell refinement: *SAINT* (Bruker, 2004 ▶); data reduction: *SAINT*; program(s) used to solve structure: *SIR2002* (Burla *et al.*, 2005 ▶); program(s) used to refine structure: *SHELXL97* (Sheldrick, 2008 ▶); molecular graphics: *ORTEP-3 for Windows* (Farrugia, 1997 ▶) and *DIAMOND* (Brandenburg & Berndt, 2001 ▶); software used to prepare material for publication: *WinGX* (Farrugia, 1999 ▶).

## Supplementary Material

Crystal structure: contains datablock(s) global, I. DOI: 10.1107/S1600536812015310/lh5448sup1.cif

Structure factors: contains datablock(s) I. DOI: 10.1107/S1600536812015310/lh5448Isup2.hkl

Supplementary material file. DOI: 10.1107/S1600536812015310/lh5448Isup3.cml

Additional supplementary materials:  crystallographic information; 3D view; checkCIF report

## Figures and Tables

**Table 1:** Hydrogen-bond geometry (Å, °)

*D*—H⋯*A*	*D*—H	H⋯*A*	*D*⋯*A*	*D*—H⋯*A*
N5—H5⋯Br3^i^	0.88	2.35	3.216 (3)	168
C6—H6*A*⋯Br2^ii^	0.96	2.90	3.796 (3)	156

Symmetry codes: (i) 
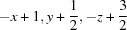
; (ii) 
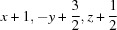
.

**Fig. 1.**
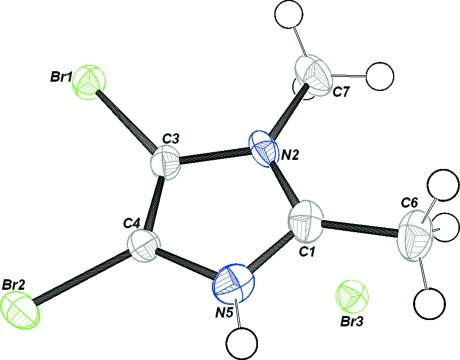
The molecular structure of the title compound with displacement ellipsoids drawn at the 50% probability level.

**Fig. 2.**
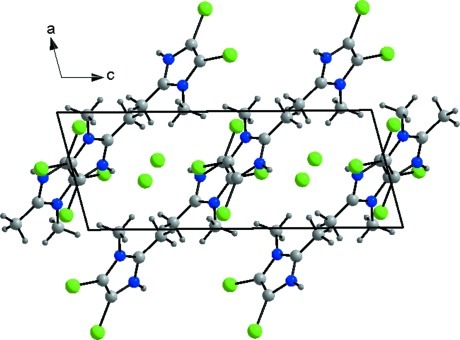
Part of the crystal structure viewed along the b axis.

**Fig. 3.**
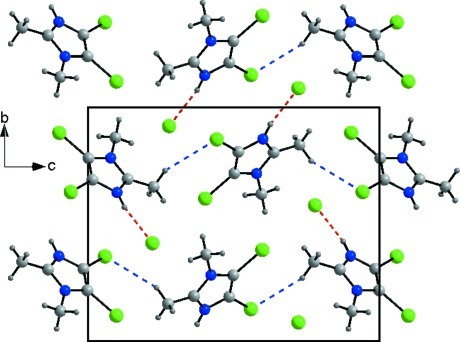
Part of the crystal structure showing hydrogen bonds [N—H···Br (in red), C—H···Br (in blue)] as dashed lines.

## supplementary crystallographic information

### Comment

Imidazole is an important synthon for the synthesis of diverse derivatives and various condensed heterocycles. The C,*N*-substituted haloimidazole derivatives have shown a high pharmacological activity (Zamora *et al.*, 2003; Schmidt *et al.*, 2003) and some have found practical use in medicine (Mashkovskii, 2005; Amini *et al.*, 2007; Reepmeyer *et al.*, 1975). Halo- and dihaloimidazoles form salts with mineral acids and picrates. The nitrates and picrates, which crystallize readily from water and alcohols, are quite often used for the additional characterization of compounds being studied. In this paper, we report the structure determination of 4,5-dibromo-1,2-dimethyl-1*H*-imidazolium bromide (I) resulting from an unexpected reaction of 1,2-dimethyl-1*H*-imidazole with bromine in acetone in a modified Ortoleva-King conditions reaction (King, 1944).

The molecular structure of (I) is shown in Fig. 1. The asymmetric unit of title molecule, (C_5_H_7_N_2_Br_2_)^+^, Br^-^, contains a 4,5-dibromo-1,2-dimethylimidazolium cation and bromide anion linked by an intermolecular N—H···Br hydrogen bond. The crystal packing can be described as intercalated layers parallel to (102) in which bromide ions are located between cations (Fig. 2). Further stabilization is provided by weak intermoleculer C—H···Br hydrogen bonds (Fig. 3).

### Experimental

Compound (I) was obtained from reaction of 4,5-dibromo-1,2-dimethyl-1*H*-imidazole dissolved in acetone with 1 eq. of bromine. After stirring at 303K during 1 h, a colorless suspension was obtained and a white solid was filtered off. A suitable crystal was obtained by slow evaporation at room temperature of a solution of (I) in a MeOH/CHCl_3_ mixture.

### Refinement

H atoms were located in difference Fourier maps but introduced in calculated positions and treated as riding on their parent C or N atom (with C—H = 0.96 Å, N—H = 0.88 Å and *U*_iso_(H) = 1.5U_eq_(C) or 1.2U_eq_(N).

### Crystal data

**Table:**

C_5_H_7_Br_2_N_2_^+^·Br^−^	*F*(000) = 624
*M**_r_* = 334.86	*D*_x_ = 2.52 Mg m^−^^3^
Monoclinic, *P*2_1_/*c*	Mo *K*α radiation, λ = 0.71073 Å
Hall symbol: -P 2ybc	Cell parameters from 3086 reflections
*a* = 5.5938 (3) Å	θ = 2.9–27.5°
*b* = 11.2522 (6) Å	µ = 13.64 mm^−^^1^
*c* = 14.4864 (9) Å	*T* = 150 K
β = 104.571 (3)°	Prism, colourless
*V* = 882.48 (9) Å^3^	0.31 × 0.22 × 0.17 mm
*Z* = 4	

### Data collection

**Table:**

Bruker APEXII diffractometer	1747 reflections with *I* > 2σ(*I*)
Graphite monochromator	*R*_int_ = 0.050
CCD rotation images, thin slices scans	θ_max_ = 27.5°, θ_min_ = 3.4°
Absorption correction: multi-scan (*SADABS*; Bruker, 2002)	*h* = −6→7
*T*_min_ = 0.058, *T*_max_ = 0.098	*k* = −14→12
7565 measured reflections	*l* = −18→17
2032 independent reflections	

### Refinement

**Table:**

Refinement on *F*^2^	Secondary atom site location: difference Fourier map
Least-squares matrix: full	Hydrogen site location: inferred from neighbouring sites
*R*[*F*^2^ > 2σ(*F*^2^)] = 0.027	H-atom parameters constrained
*wR*(*F*^2^) = 0.060	*w* = 1/[σ^2^(*F*_o_^2^) + (0.0201*P*)^2^ + 0.142*P*] where *P* = (*F*_o_^2^ + 2*F*_c_^2^)/3
*S* = 1.03	(Δ/σ)_max_ = 0.002
2032 reflections	Δρ_max_ = 0.63 e Å^−^^3^
94 parameters	Δρ_min_ = −0.86 e Å^−^^3^
0 restraints	Extinction correction: *SHELXL97* (Sheldrick, 2008), Fc^*^=kFc[1+0.001xFc^2^λ^3^/sin(2θ)]^-1/4^
Primary atom site location: structure-invariant direct methods	Extinction coefficient: 0.0075 (4)

### Special details

**Table:**

Geometry. All e.s.d.'s (except the e.s.d. in the dihedral angle between two l.s. planes) are estimated using the full covariance matrix. The cell e.s.d.'s are taken into account individually in the estimation of e.s.d.'s in distances, angles and torsion angles; correlations between e.s.d.'s in cell parameters are only used when they are defined by crystal symmetry. An approximate (isotropic) treatment of cell e.s.d.'s is used for estimating e.s.d.'s involving l.s. planes.
Refinement. Refinement of *F*^2^ against ALL reflections. The weighted *R*-factor *wR* and goodness of fit *S* are based on *F*^2^, conventional *R*-factors *R* are based on *F*, with *F* set to zero for negative *F*^2^. The threshold expression of *F*^2^ > σ(*F*^2^) is used only for calculating *R*-factors(gt) *etc*. and is not relevant to the choice of reflections for refinement. *R*-factors based on *F*^2^ are statistically about twice as large as those based on *F*, and *R*- factors based on ALL data will be even larger.

### Fractional atomic coordinates and isotropic or equivalent isotropic displacement parameters (Å^2^)

**Table:**

	*x*	*y*	*z*	*U*_iso_*/*U*_eq_	
Br1	0.52217 (6)	0.60967 (3)	0.40218 (2)	0.01621 (11)	
Br2	0.12689 (6)	0.86245 (3)	0.44145 (2)	0.01726 (11)	
N2	0.7783 (5)	0.7167 (2)	0.57813 (19)	0.0142 (6)	
N5	0.5520 (5)	0.8641 (3)	0.5998 (2)	0.0171 (6)	
H5	0.4987	0.9266	0.6251	0.021*	
C1	0.7626 (6)	0.8069 (3)	0.6365 (2)	0.0163 (7)	
C3	0.5709 (6)	0.7178 (3)	0.5019 (2)	0.0141 (7)	
C4	0.4301 (6)	0.8096 (3)	0.5154 (2)	0.0140 (7)	
C6	0.9445 (7)	0.8397 (3)	0.7253 (2)	0.0222 (8)	
H6A	0.9879	0.7706	0.7647	0.033*	
H6B	0.8745	0.8986	0.7587	0.033*	
H6C	1.0896	0.8713	0.7103	0.033*	
C7	0.9765 (7)	0.6294 (3)	0.5945 (3)	0.0214 (8)	
H7A	1.1062	0.6531	0.6483	0.032*	
H7B	1.0403	0.6242	0.5390	0.032*	
H7C	0.9137	0.5532	0.6069	0.032*	
Br3	0.58756 (6)	0.58078 (3)	0.77871 (2)	0.01713 (12)	

### Atomic displacement parameters (Å^2^)

**Table:**

	*U*^11^	*U*^22^	*U*^33^	*U*^12^	*U*^13^	*U*^23^
Br1	0.0170 (2)	0.01589 (19)	0.01515 (19)	0.00097 (14)	0.00295 (14)	−0.00101 (12)
Br2	0.01324 (19)	0.0185 (2)	0.01989 (19)	0.00293 (14)	0.00389 (14)	0.00221 (13)
N2	0.0086 (14)	0.0175 (15)	0.0156 (14)	−0.0021 (12)	0.0013 (11)	0.0037 (11)
N5	0.0193 (16)	0.0141 (15)	0.0185 (15)	−0.0040 (12)	0.0060 (12)	−0.0026 (11)
C1	0.0153 (18)	0.0171 (18)	0.0162 (17)	−0.0055 (14)	0.0031 (14)	0.0020 (14)
C3	0.0124 (17)	0.0172 (17)	0.0123 (16)	−0.0017 (14)	0.0022 (13)	0.0011 (13)
C4	0.0107 (17)	0.0173 (18)	0.0138 (16)	−0.0015 (14)	0.0029 (13)	−0.0020 (13)
C6	0.021 (2)	0.025 (2)	0.0182 (18)	−0.0087 (16)	0.0008 (15)	−0.0019 (15)
C7	0.0156 (19)	0.023 (2)	0.024 (2)	0.0058 (15)	0.0019 (16)	0.0075 (14)
Br3	0.0163 (2)	0.0161 (2)	0.01874 (19)	−0.00155 (13)	0.00405 (14)	−0.00052 (13)

### Geometric parameters (Å, º)

**Table:**

Br1—C3	1.855 (3)	C1—C6	1.472 (5)
Br2—C4	1.861 (3)	C3—C4	1.343 (5)
N2—C1	1.338 (4)	C6—H6A	0.9600
N2—C3	1.386 (4)	C6—H6B	0.9600
N2—C7	1.456 (4)	C6—H6C	0.9600
N5—C1	1.329 (4)	C7—H7A	0.9600
N5—C4	1.385 (4)	C7—H7B	0.9600
N5—H5	0.8800	C7—H7C	0.9600
			
C1—N2—C3	108.8 (3)	N5—C4—Br2	122.8 (2)
C1—N2—C7	125.3 (3)	C1—C6—H6A	109.5
C3—N2—C7	125.9 (3)	C1—C6—H6B	109.5
C1—N5—C4	109.2 (3)	H6A—C6—H6B	109.5
C1—N5—H5	125.4	C1—C6—H6C	109.5
C4—N5—H5	125.4	H6A—C6—H6C	109.5
N5—C1—N2	107.9 (3)	H6B—C6—H6C	109.5
N5—C1—C6	125.2 (3)	N2—C7—H7A	109.5
N2—C1—C6	127.0 (3)	N2—C7—H7B	109.5
C4—C3—N2	107.1 (3)	H7A—C7—H7B	109.5
C4—C3—Br1	129.9 (2)	N2—C7—H7C	109.5
N2—C3—Br1	123.0 (2)	H7A—C7—H7C	109.5
C3—C4—N5	107.0 (3)	H7B—C7—H7C	109.5
C3—C4—Br2	130.2 (2)		
			
C4—N5—C1—N2	−0.3 (4)	C1—N2—C3—Br1	178.4 (2)
C4—N5—C1—C6	179.1 (3)	C7—N2—C3—Br1	−3.8 (5)
C3—N2—C1—N5	0.4 (4)	N2—C3—C4—N5	0.0 (4)
C7—N2—C1—N5	−177.4 (3)	Br1—C3—C4—N5	−178.5 (2)
C3—N2—C1—C6	−179.0 (3)	N2—C3—C4—Br2	−179.7 (2)
C7—N2—C1—C6	3.2 (5)	Br1—C3—C4—Br2	1.7 (5)
C1—N2—C3—C4	−0.2 (4)	C1—N5—C4—C3	0.2 (4)
C7—N2—C3—C4	177.5 (3)	C1—N5—C4—Br2	180.0 (2)

### Hydrogen-bond geometry (Å, º)

**Table:**

*D*—H···*A*	*D*—H	H···*A*	*D*···*A*	*D*—H···*A*
N5—H5···Br3^i^	0.88	2.35	3.216 (3)	168
C6—H6*A*···Br2^ii^	0.96	2.90	3.796 (3)	156

Symmetry codes: (i) −*x*+1, *y*+1/2, −*z*+3/2; (ii) *x*+1, −*y*+3/2, *z*+1/2.
